# Patterns and Predictors of Breast Milk Feeding from Birth to Age 4 Months among Primiparous African American Mother–Infant Dyads

**DOI:** 10.3390/nu14112350

**Published:** 2022-06-04

**Authors:** Amy M. Moore, Jessica J. Smith, Brian K. Stansfield, Jennifer S. Savage, Justin A. Lavner

**Affiliations:** 1 Center for Childhood Obesity Research, The Pennsylvania State University, 129 Noll Laboratory, University Park, PA 16802, USA; jfs195@psu.edu; 2 Center for Family Research, University of Georgia, Athens, GA 30602, USA; jjsmith@uga.edu; 3 Department of Pediatrics, Medical College of Georgia, Augusta University, Augusta, GA 30912, USA; bstansfield@augusta.edu; 4 Department of Psychology, University of Georgia, 156 Psychology Building, Athens, GA 30602, USA; lavner@uga.edu

**Keywords:** breastfeeding, breast milk, human milk, infant feeding, African American, Sleep SAAF

## Abstract

The health benefits of breast milk feeding have been well-established, yet disparities exist, with African American mothers having the lowest breast milk feeding rates in the United States. This prospective, longitudinal study examined infant feeding (breast milk and/or infant formula) from birth to age 16 weeks, predictors of any breast milk feeding by age 1 week, and predictors of cessation of any breast milk feeding by ages 3, 8, and 16 weeks among primiparous African American mothers. This secondary analysis included 185 mother–infant dyads from the Sleep SAAF (Strong African American Families) study, a randomized clinical trial testing a responsive parenting vs. child safety control intervention. Mothers reported sociodemographic and psychosocial characteristics at age 1 week and infant feeding practices at ages 1, 3, 8, and 16 weeks. Rates of any breast milk feeding decreased from 66.5% at 1 week to 23.3% at 16 weeks. Bivariate logistic regression models showed that prepregnancy BMI (OR = 1.09), working prepregnancy (OR = 2.25), and food insecurity (OR = 2.49) significantly increased the odds of mothers feeding any breast milk by 1 week, whereas Special Supplemental Nutrition Program for Women, Infants, and Children (WIC) participation (OR = 0.21) significantly decreased the odds. Bivariate logistic regression models showed that Supplemental Nutrition Assistance Program (SNAP) participation (OR = 2.86) and racial discrimination (OR = 2.14) significantly increased the odds of cessation of any breast milk feeding by 3 weeks. SNAP (OR = 2.33) and WIC (OR = 2.38) participation significantly increased the odds of cessation of any breast milk feeding by 8 weeks, whereas higher prepregnancy BMI (OR = 0.95) decreased the odds. Higher mother’s age (OR = 0.92) significantly decreased the odds of cessation of any breast milk feeding by 16 weeks. The findings can be used to inform targeted interventions to promote mothers feeding any breast milk and help reduce breast milk feeding disparities among African American mothers.

## 1. Introduction

Infant feeding practices, including what and how infants are fed, play important roles in early growth and development. The Dietary Guidelines for Americans [1] and the American Academy of Pediatrics [2] recommend exclusive breast milk feeding (defined as feeding only breast milk, including expressed breast milk) through age 6 months, with continued breast milk feeding while introducing complementary foods through at least age 12 months. The health benefits of breastfeeding for both mother and infant are well-established. For mothers, breastfeeding reduces the risk for hypertension, type 2 diabetes mellitus, some cancers, and postpartum depression [3,4,5,6]. For infants, along with providing essential nutrition, breastfeeding reduces the risk for gastroenteritis, otitis media, and sudden infant death syndrome [5,7]. Despite these and other health benefits, national data reveal disparities in breast milk feeding initiation, exclusivity, and continuation rates, with African American mothers having the lowest breast milk feeding rates in the United States (US) [8,9,10]. African American mothers report lower exclusive breast milk feeding at infant ages 3 months (39% vs. 51%) and 6 months (20% vs. 29%) when compared to White mothers, with generally lower rates reported in the southeastern US [11]. As such, infant formula feeding is higher among African American mother–infant dyads [8,9,10]. Understanding infant feeding practices among African American mothers, including what (breast milk and/or infant formula feeding) and how (bottle-feeding practices) infants are fed, and identifying factors that influence breast milk feeding are essential to reduce disparities in breast milk feeding and better promote optimal infant growth and development.

A number of sociodemographic characteristics have been associated with higher rates of exclusive breast milk feeding in nationally representative samples, including higher mother’s age, educational attainment, and household income, as well as married relationship status [12,13]. Conversely, single-caregiver households, a history of anxiety or depression during pregnancy, and early return to work have been associated with lower rates of exclusive breast milk feeding [12,13,14,15]. Previous research also suggests that African American mothers disproportionately experience structural barriers and inequities related to breast milk feeding, such as a lack of social support, inadequate messaging and support from health care providers, as well as concerns about continued breast milk feeding with early return to work [16,17]. However, previous research has largely focused on exclusive breast milk feeding and longer duration (i.e., ages 6 and 12 months), while less is known about the predictors of any breast milk feeding and early cessation. In addition, interpreting previous research is often hampered by comparisons with White mothers rather than within-group comparisons, which can be used to understand the unique factors that influence breast milk feeding among African American mothers.

To address these gaps, the current study used longitudinal data from a sample of primiparous African American mothers residing in the southeastern US to understand infant feeding practices, including what (breast milk and/or infant formula) mothers fed their infants, at ages 1, 3, 8, and 16 weeks. Repeated assessment of mothers’ infant feeding practices is particularly valuable to understand how these practices change over time and whether there are sensitive periods when these practices change. This study also examined prospective associations between sociodemographic and psychosocial characteristics and any breast milk versus exclusive infant formula feeding by age 1 week to understand which subgroups of mothers were more likely to feed any breast milk. Prospective associations between these sociodemographic and psychosocial characteristics and cessation of any breast milk feeding versus continuation by ages 3, 8, and 16 weeks were also examined to understand which subgroups of mothers were more likely to stop feeding any breast milk.

## 2. Materials and Methods

### 2.1. Study Design and Participants

This was a secondary analysis of data from the Sleep SAAF (Strong African American Families) study, a two-arm randomized clinical trial that included 212 primiparous African American mothers and their newborn infants residing in the southeastern US [18]. The Sleep SAAF study was designed to evaluate the effects of a responsive parenting (RP) intervention, adapted from the INSIGHT RP intervention [19], on rapid infant weight gain during the first 16 weeks of life compared to a child safety control intervention. Mother–infant dyads were recruited shortly after delivery (infant M_age_ = 1.5 days at enrollment) from a mother/baby nursery in Georgia from 2018 to 2021. Primiparous mothers ≥ 17 years of age were eligible if they self-identified as African American/Black, had a full-term (≥37 weeks gestational age) singleton pregnancy, were English speaking, lived within ≤75 miles of Augusta, and had an infant ≥ 2500 g at birth. Dyads were excluded if the mother had a known medical condition that could impact postnatal care (e.g., serious mental illness, substance use disorder), if the infant had a known medical condition that would impact feeding or growth (e.g., cleft palate), if there was an adoption plan in place, or if there was a plan to move out of the area within four months of delivery. Details on the study design have been published elsewhere [18]. Mothers provided written informed consent and parental permission for their infants during enrollment. Sleep SAAF was approved by the Augusta University Institutional Review Board and was registered on www.clinicaltrials.gov (NCT03505203), accessed on 15 November 2021.

### 2.2. Procedures

Trained Community Research Associates (CRAs), who were local community members who self-identified as African American/Black, conducted home visits approximately at infant ages 1, 3, 8, and 16 weeks. At each time point, mothers completed online sociodemographic, psychosocial, and infant feeding questionnaires using Qualtrics. At infant ages 3 and 8 weeks, mothers participated in an RP intervention that provided guidance on infant sleeping, crying, feeding, and interactive play or a child safety control intervention.

### 2.3. Measures

#### 2.3.1. Sociodemographic Characteristics

Mothers’ ages and self-identified race, along with infant sex and gestational age, were obtained from electronic medical records. At enrollment, mothers’ heights (Seca 274, Hanover, MD, USA) were measured in duplicate (or triplicate if the first two measurements differed by more than 0.2 cm) by trained research staff, and mothers’ self-reported prepregnancy weights were recorded. Measured height and self-reported prepregnancy weight were used to calculate prepregnancy body mass index (BMI). Infant recumbent length (Seca 416 Infantometer, Hanover, MD, USA) and weight (Medela BabyWeigh II Scale, McHenry, IL, USA) were measured in duplicate (or triplicate if the first two measurements differed by more than 0.2 cm or 50 g, respectively) at enrollment. These measurements were used to calculate sex-specific weight-for-length z-scores (WFLz) at enrollment using World Health Organization (WHO) reference standards [20].

Mothers reported sociodemographic characteristics at infant age 1 week, including romantic relationship status, employment status prepregnancy, annual household income, highest education level, the total number in household, and participation in the Supplemental Nutrition Assistance Program (SNAP) and the Special Supplemental Nutrition Program for Women, Infants, and Children (WIC). A validated two-item screening tool designed to identify families at risk for food insecurity (i.e., a lack of consistent access to enough food for a healthy, active life) was also completed by mothers at age 1 week and used to categorize families as food insecure (yes or no) during the previous 12 months [21]. Mothers also reported their employment status at infant ages 8 and 16 weeks.

#### 2.3.2. Psychosocial Characteristics

Mothers reported psychosocial characteristics at infant age 1 week. Depressive symptoms during the previous seven days were assessed using the 20-item Center for Epidemiological Studies Depression Scale (CES-D) [22]. Response options ranged from 0 (rarely or none of the time) to 3 (most or all of the time). Items were averaged to produce a mean score, with higher scores indicating greater depressive symptoms (α = 0.84) [22]. For mothers in a current romantic relationship, relationship satisfaction was assessed using three items from the Couples Satisfaction Index (CSI) [23]. Response options ranged from 1 (extremely unhappy) to 7 (perfect) or 1 (not at all) to 6 (completely). Items were summed to produce a total score, with higher scores indicating greater relationship satisfaction (α = 0.69). Social support was assessed using two subscales (reliable alliance and guidance) from the 24-item Social Provisions Scale (SPS) [24]. Reliable alliance captures the degree to which respondents perceive that there are people in their lives who can be counted on for support during times of stress (four items; e.g., “There are people I can depend on to help me if I really need it”; α = 0.66). Guidance captures the degree to which respondents perceive that there are people in their lives who can be counted on for advice or information during times of stress (four items; e.g., “There is a trustworthy person I could turn to for advice if I were having problems”; α = 0.68). Response options ranged from 1 (strongly disagree) to 4 (strongly agree). Items were averaged to produce a mean score, with higher scores indicating greater social support. Racial discrimination was assessed using the 17-item Schedule of Racist Events (SRE) [25]. This measure captures the frequency of discriminatory behaviors during the past year, including unfair treatment, racially based slurs, and physical threats (e.g., “How many times in the past year have you been called a racist name?”). Response options ranged from 1 (never) to 6 (almost all of the time). Items were averaged to produce a mean score, with higher scores indicating higher levels of racial discrimination in the past year (α = 0.93).

#### 2.3.3. Assessment of Infant Feeding Practices

Mothers reported developmentally relevant infant feeding practices at infant ages 1, 3, 8, and 16 weeks. Mothers were asked about infant feeding (breast milk and/or infant formula) at age 1 week. Items from the Babies Need Feeding component of the Baby’s Basic Needs Questionnaire were used to assess infant feeding (breast milk and/or infant formula) at ages 3, 8, and 16 weeks [26]. Infant feeding at each time point was categorized as “exclusive breast milk” (defined as feeding breast milk only), “partial breast milk” (defined as feeding a combination of breast milk and infant formula), and “exclusive infant formula” (defined as feeding infant formula only) feeding. Items from Babies Need Feeding were also used to assess bottle-feeding practices (i.e., bottle and nipple size, adding cereal to the bottle, and adding beverages other than breast milk or infant formula to the bottle) for mothers who reported any infant formula feeding at ages 3, 8, and 16 weeks [26].

### 2.4. Statistical Analyses

Descriptive statistics, including means and standard deviations for continuous variables and frequencies and percentages for categorical variables, were used to summarize the main variables of interest. This analysis included 185 mother–infant dyads for which there were complete data on infant feeding at infant ages 1, 3, 8, and 16 weeks. Bivariate logistic regression models examined associations between sociodemographic and psychosocial characteristics and any breast milk (reference category) versus exclusive infant formula feeding by age 1 week. Bivariate logistic regression models also examined associations between sociodemographic and psychosocial characteristics and cessation of any breast milk feeding (reference category) versus continuation by ages 3, 8, and 16 weeks. For this analysis, the predictor variable “in a romantic relationship” was created by collapsing “married, living together”, “living together”, and “involved, but not living together”, and the predictor variable “currently working outside the home” was based on infant age when mothers returned to work after delivery. The outcome variable “any breast milk” (defined as feeding any breast milk) was created by collapsing “exclusive breast milk” and “partial breast milk” at each time point separately. Any breast milk was used as the reference category given the health benefits of any breast milk feeding for mothers and infants [27]. Main variables of interest were examined by study group (RP vs. child safety control intervention) at ages 8 and 16 weeks (the two post-intervention time points) using independent-sample *t*-tests for continuous variables and chi-squared tests for categorical variables. All models were initially run adjusting for study group; however, as this did not significantly alter the pattern of results, final models were not adjusted for study group. Data were analyzed using SAS 9.4 [28] and an alpha of *p* < 0.05 (two-tailed) was used to determine statistical significance.

## 3. Results

### 3.1. Sociodemographic Characteristics

Table 1 presents sociodemographic and psychosocial characteristics of mother–infant dyads. Mothers self-identified as African American/Black (100%) and were on average 22.9 (SD = 4.6) years of age; 61.1% were in a romantic relationship, 50.3% were working full-time or part-time prepregnancy, and 76.4% participated in WIC.

### 3.2. Infant Feeding Practices

**Figure 1:**
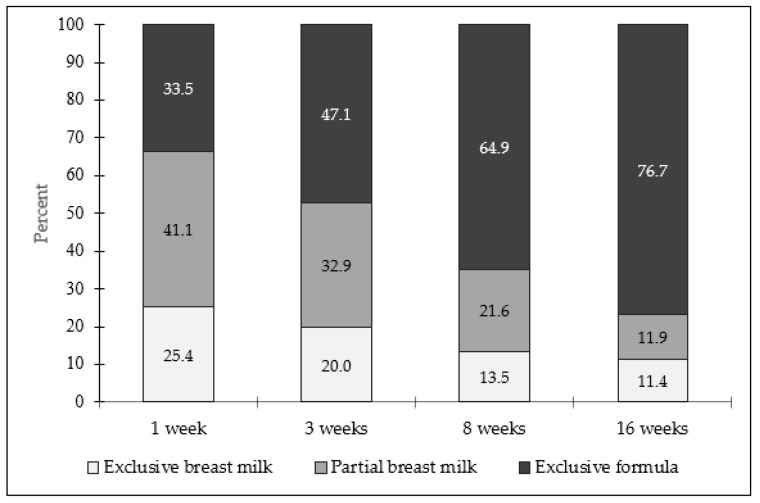
Mothers’ infant feeding at ages 1, 3, 8, and 16 weeks (*n* = 185). Infant feeding defined as “exclusive breast milk” (feeding breast milk only), “partial breast milk” (feeding a combination of breast milk and infant formula), and “exclusive infant formula” (feeding infant formula only).

Figure 1 presents infant feeding at infant ages 1, 3, 8, and 16 weeks. Rates of exclusive and partial breast milk feeding decreased over the course of the study. At 1 week, 25.4% of mothers reported “exclusive breast milk” feeding, which declined to 20.0% at 3 weeks, 13.5% at 8 weeks, and 11.4% at 16 weeks. Similarly, 41.1% of mothers reported “partial breast milk” feeding at 1 week, which declined to 32.9% at 3 weeks, 21.6% at 8 weeks, and 11.9% at 16 weeks. Collectively, rates of any breast milk feeding decreased from 66.5% at 1 week to 52.9% at 3 weeks, 35.1% at 8 weeks, and 23.3% at 16 weeks. Of the mothers reporting any breast milk feeding, 14.3% reported feeding expressed breast milk at 3 weeks, 84.6% at 8 weeks, and 76.7% at 16 weeks. Rates of “exclusive infant formula” feeding increased over the course of the study, ranging from 33.5% at 1 week to 76.7% at 16 weeks.

### 3.3. Associations between Sociodemographic and Psychosocial Characteristics and Any Breast Milk Feeding by Infant Age 1 Week

Table 2 presents results for bivariate logistic regression models for associations between select sociodemographic and psychosocial characteristics with any breast milk feeding at infant age 1 week. Higher prepregnancy BMI (OR = 1.09), working prepregnancy (OR = 2.25), and food insecurity (OR = 2.49) significantly increased the odds of any breast milk feeding by age 1 week, whereas WIC participation (OR = 0.21) decreased the odds. Psychosocial characteristics (e.g., depressive symptoms, relationship satisfaction, racial discrimination) were not significant predictors of any breast milk feeding by age 1 week.

### 3.4. Associations between Sociodemographic and Psychosocial Characteristics and Cessation of Any Breast Milk Feeding by Infant Ages 3, 8, and 16 Weeks

Table 3 presents results for bivariate logistic regression models for associations between select sociodemographic and psychosocial characteristics and cessation of any breast milk feeding by infant ages 3, 8, and 16 weeks. SNAP participation (OR = 2.86) and racial discrimination (OR = 2.14) significantly increased the odds of cessation of any breast milk feeding by age 3 weeks. SNAP (OR = 2.33) and WIC (OR = 2.38) participation significantly increased the odds of cessation of any breast milk feeding by age 8 weeks, whereas higher prepregnancy BMI (OR = 0.95) significantly decreased the odds. Higher mothers’ age (OR = 0.92) significantly decreased the odds of cessation of any breast milk feeding by age 16 weeks. There were no other significant predictors of cessation of any breast milk feeding at any time point.

### 3.5. Bottle-Feeding Practices

Given the large and growing subgroup of mothers feeding infant formula, bottle-feeding practices among mothers who reported feeding any infant formula (i.e., “exclusive infant formula” and “partial breast milk”) were examined at infant ages 3, 8, and 16 weeks. Most mothers reported using a developmentally appropriate bottle size at 3 (82.1%), 8 (72.7%), and 16 (75.0%) weeks. Similarly, most mothers reported using a developmentally appropriate nipple size at 3 (79.1%), 8 (69.7%), and 16 (97.4%) weeks. However, a growing number of mothers reported adding cereal to their infant’s bottle from 3 (8.1%), 8 (22.5%), and 16 (50.0%) weeks. Similarly, some mothers reported adding beverages other than breast milk or infant formula to their infant’s bottle from 3 (18.2%), 8 (22.5%), and 16 (21.9%) weeks.

## 4. Discussion

The present study describes infant feeding practices among primiparous African American mothers living in the southeastern US. Given the health benefits of breast milk, this study also examined predictors of any breast milk feeding by infant age 1 week and predictors of cessation of any breast milk feeding by ages 3, 8, and 16 weeks. Findings show that rates of any breast milk feeding (i.e., exclusive and partial breast milk feeding) decreased from birth to 16 weeks, while rates of exclusive infant formula feeding increased. Findings also suggest that sociodemographic and psychosocial characteristics were differentially associated with any breast milk feeding, as well as cessation, by age 16 weeks. How these data can be used to inform targeted interventions that promote breast milk feeding and potentially reduce health disparities among African American mothers and their infants will be discussed.

Disparities exist in breast milk feeding, with African American mothers having the lowest rates of breast milk feeding compared to other racial and ethnic groups in the US [8,9,10]. Rates of exclusive and any breast milk feeding in this study were lower than national rates previously reported among African American mothers, and cessation of exclusive breast milk feeding typically occurred earlier than recommended (i.e., infant age 6 months) among mothers in this study [1,2]. Rates of exclusive breast milk feeding decreased from 25.4% at age 1 week to 11.4% at age 16 weeks, which is lower than the national rate of 19.8% at age 6 months among African American mothers [11]. Similarly, rates of any breast milk feeding decreased from 66.5% at age 1 week to 23.3% at age 16 weeks, which is lower than the national rate of 49.3% at age 6 months among African American mothers [11]. Current findings are consistent with other studies suggesting that African American mothers, particularly mothers residing in the southeastern US [29], may experience additional structural barriers and inequities exacerbating disparities in breast milk feeding [30]. Although most mothers were feeding any breast milk at age 1 week, sharp decreases in any breast milk feeding by age 16 weeks suggest that additional efforts are needed to support breast milk feeding among African American mothers.

Although exclusive breast milk feeding is widely accepted as the gold standard of nutrition for most infants for the first 6 months [1,2], any breast milk feeding confers health benefits [31]. However, previous research has primarily focused on predictors of exclusive breast milk feeding; therefore, less is known about predictors of any breast milk feeding, particularly among African American mothers. Current findings suggest that sociodemographic characteristics were differentially associated with any breast milk versus exclusive infant formula feeding by age 1 week. Specifically, mothers with higher prepregnancy BMI and who experienced food insecurity during the previous 12 months were more likely to report any breast milk feeding, whereas mothers participating in the WIC were less likely. Two of these findings are inconsistent with previous research suggesting that mothers with overweight and obesity [32] and mothers who experience food insecurity [33] are less likely to report exclusive breast milk feeding. This inconsistency may be partially explained by differences in how infant feeding was categorized in the current study (i.e., any breast milk) versus prior studies (i.e., exclusive breast milk), further highlighting the importance of examining these categories separately. Consistent with previous research [34], WIC participants in this study were less likely to report any breast milk feeding and were more likely to report early cessation. Although breast milk feeding support is a priority of the WIC program, and rates of breast milk feeding have increased among WIC-participating mother–infant dyads in recent decades, rates of breast milk feeding at age 6 months continue to be lower among WIC participants compared to WIC-eligible nonparticipants [35]. Further research into these patterns and identifying how federal nutrition assistance programs, such as the WIC, can help improve breast feeding rates and reduce disparities among African American mothers is needed.

This study also adds to a small body of research showing that racial discrimination plays a role in breast milk feeding decisions among African American mothers [36]. Current findings indicate that mothers experiencing higher levels of racial discrimination over the previous year are more likely to report cessation of any breast milk feeding by age 3 weeks, supporting the idea that larger societal contexts such as structural racism may influence breast milk feeding disparities and health inequities [37]. As such, Petit et al. call for providers working with pregnant and breast milk feeding women to understand how these broader social contexts impact breast milk feeding decisions among African American women and for the development of equity-based federal policies to reduce breast milk feeding disparities [30]. In addition, although marginally but not statistically significant, older mothers were more likely to report any breast milk feeding at 1 week and less likely to report cessation of breast milk feeding by age 3 and 8 weeks. By age 16 weeks, older mothers were significantly less likely to report cessation of any breast milk feeding. Together these findings suggest that younger mothers may benefit from additional support to improve rates of breast milk feeding. Surprisingly, psychosocial characteristics, such as depressive symptoms and romantic relationship satisfaction, did not predict any breast milk feeding in this sample. Previous research has primarily compared these characteristics between racial and ethnic groups [12,13], and thus there was a need to compare these characteristics among African American women. Additional research is needed to understand how these factors influence feeding practices among African American mothers from various socioeconomic backgrounds and geographic regions to better understand their breast milk feeding experiences.

Exclusive infant formula feeding was higher than expected and increased throughout the study. Understanding bottle-feeding practices among African American mothers is particularly important since suboptimal bottle-feeding practices can promote overfeeding [38]. For this study, questions related to bottle-feeding were only asked of mothers who reported any infant formula feeding, and thus bottle-feeding practices of mothers who exclusively fed infants expressed breast milk are unknown. Nonetheless, most mothers reported using developmentally appropriate bottle and nipple sizes, which has been associated with healthy infant growth [38]. However, in contrast to infant feeding recommendations [1,2], some mothers reported adding cereal and beverages to their infant’s bottle, which is concerning given that these practices have been associated with overfeeding and rapid infant weight gain [39]. These findings suggest additional support related to developmentally appropriate bottle-feeding practices is warranted. Previous studies of bottle-feeding practices suggest that mothers add cereal or beverages to their infant’s bottle for a variety of reasons, such as to soothe or calm their infant’s distress or help their infant sleep [38], which are not recommended but may help explain these findings. Additional research is needed to understand bottle-feeding practices among African American mothers regardless of what they choose to feed their infant (breast milk and/or infant formula). Future interventions designed to promote optimal feeding practices during early infancy should include tailored messaging related to optimal bottle-feeding practices to help mothers meet infant feeding recommendations throughout early infancy.

Although this study has many strengths, including examining infant feeding (breast milk and/or infant formula) and predictors of any breast milk feeding and cessation of any breast milk feeding at multiple time points during early infancy, it is not without limitations. This sample included primiparous African American mothers residing in the southeastern US who were recruited from a single hospital, which limits generalizability. However, this study recruited mother–infant dyads from a large hospital in the southeastern US serving a rural area, and African American mothers residing in this region have lower rates of breast milk feeding, which makes understanding infant feeding in this population important [11]. Additionally, the current study did not assess reasons for and influences on mothers’ early infant feeding decisions. Future studies should examine these factors to better understand how mothers make early infant feeding decisions. This study did not assess bottle-feeding practices at infant age 1 week or for mothers who reported exclusively feeding their infant expressed breast milk. Future studies should assess bottle-feeding practices for all mothers regardless of what (i.e., expressed breast milk and/or infant formula) they choose to feed their infant. This study also did not assess the proportion of breast milk versus infant formula feeding, so the quantity of breast milk infants received is unknown. In addition, many of the characteristics included in this study may change over time; therefore, future studies should examine these factors multiple times across the first year of life to better understand the influences on breast milk feeding among African American mothers. Other factors that were not measured, such as social and cultural norms, as well as advice and support from friends and family and education from health care providers, likely play a role in any breast milk and/or infant formula feeding [40] and should be studied in future work.

## 5. Conclusions

Findings from this study reveal low exclusive breast milk feeding rates and decreases in any breast milk feeding in tandem with increases in exclusive formula feeding from birth to infant age 4 months among primiparous African American mothers from the southeastern US. Findings also suggest that any breast milk feeding and cessation were more closely associated with sociodemographic compared to psychosocial characteristics in this sample. Additional research is needed to understand how broader societal and structural contexts influence breast milk feeding among African American mothers over time and to support African American women who wish to breast milk feed.

## Figures and Tables

**Table 1:** Characteristics of mother–infant dyads (*n* = 185).

Characteristic	*n*	
Mother		
Age (years), mean (SD)	185	22.9 (4.6)
Race, African American/Black, *n* (%)	185	185 (100.0)
Prepregnancy BMI, mean (SD)	184	28.3 (8.4)
Romantic relationship status, *n* (%)	185	
Single		71 (38.4)
Married, living together		22 (11.9)
Married, but not living together		1 (0.5)
Living together		58 (31.4)
Involved, but not living together		33 (17.8)
Working prepregnancy, *n* (%)	185	
Full-time		64 (34.6)
Part-time		29 (15.7)
Student		9 (4.9)
Unemployed		80 (43.2)
Other		3 (1.6)
Currently working outside the home, *n* (%)	185	
1 week		0 (0.0)
3 weeks		19 (10.3)
8 weeks		74 (40.0)
16 weeks		103 (55.7)
Annual household income, *n* (%)	184	
<$10,000		41 (22.8)
$10,000–24,999		23 (12.5)
$25,000–49,999		26 (14.3)
$50,000 or more		16 (8.7)
Don’t know		72 (39.1)
Refuse to answer		6 (3.3)
Education, *n* (%)	185	
Some high school		22 (11.9)
High school graduate		91 (49.1)
Some college or technical school		48 (26.0)
College graduate or graduate degree		24 (13.0)
Number in household, mean (SD)	139	2.0 (1.5)
Federal Nutrition Assistance, *n* (%)		
SNAP participant	179	84 (46.9)
WIC participant	182	139 (76.4)
Food insecurity, *n* (%)		80 (43.2)
Depressive symptoms, mean (SD)	184	0.64 (0.45)
Romantic relationship satisfaction, mean (SD)	114	16.1 (2.8)
Social provision, mean (SD)	185	
Reliable alliance		3.4 (0.53)
Guidance		3.1 (0.56)
Racial discrimination, mean (SD)	185	1.4 (0.58)
**Infant**		
Male, *n* (%)	185	89 (48.1)
Gestational age (weeks), mean (SD)	185	39.1 (1.1)
Weight (kg) at enrollment, mean (SD)	185	6.0 (0.35)
Length (cm) at enrollment, mean (SD)	185	48.7 (1.7)
WFLz at enrollment, mean (SD)	182	−0.34 (0.96)

Note: BMI, body mass index; SNAP, Supplemental Nutrition Assistance Program; WIC, Special Supplemental Nutrition Program for Women, Infants, and Children; WFLz, weight-for-length z-score.

**Table 2:** Bivariate logistic regression models for associations between select sociodemographic and psychosocial characteristics and any breast milk feeding at infant age 1 week among African American mothers (*n* = 185).

	1 Week	
Characteristics ^a^	OR	95% CI
**Sociodemographic**		
Age (years)	1.08	1.00–1.16
Prepregnancy BMI	**1.09** *	1.04–1.15
In a romantic relationship ^b^	1.53	0.82–2.86
Worked outside the home prepregnancy ^c^	**2.25** *	1.21–4.18
Number in household	1.01	0.77–1.29
SNAP participation	0.55	0.30–1.04
WIC participation	**0.21** *	0.08–0.56
Food insecurity	**2.49** *	1.29–4.77
Infant WFLz at enrollment	0.75	0.54–1.05
**Psychosocial**		
Depressive symptoms	1.17	0.58–2.33
Relationship satisfaction	1.00	0.87–1.16
Social provision		
Reliable alliance	1.07	0.61–1.89
Guidance	1.30	0.74–2.28
Racial discrimination	1.16	0.67–2.00

^a^ All sociodemographic and psychosocial characteristics measured at infant age 1 week. ^b^ Includes “married, living together” and “involved, but not living together”. ^c^ Includes “full-time” and “part-time” employment outside of the home prepregnancy. Reference group for the outcome variable was “any breast milk” (*n* = 123) versus “exclusive infant formula” (*n* = 62) feeding at age 1 week. OR, odds ratio; CI, confidence interval; SNAP, Supplemental Nutrition Assistance Program; WIC, Special Supplemental Nutrition Program for Women, Infants, and Children; * WFLz, weight-for-length; *p* < 0.05 (two-tailed), significant results are bolded for emphasis.

**Table 3:** Bivariate logistic regression models for associations between select sociodemographic and psychosocial characteristics and cessation of any breast milk feeding at infant ages 3, 8, and 16 weeks among African American mothers (*n* = 185).

	3 Weeks		8 Weeks		16 Weeks	
Characteristics ^a^	OR	95% CI	OR	95% CI	OR	95% CI
Sociodemographic						
Age (years)	0.90	0.81–1.00	0.93	0.87–1.00	0.92 *	0.85–0.98
Prepregnancy BMI	0.97	0.92–1.03	0.95 *	0.91–0.99	1.01	0.96–1.05
In a romantic relationship ^b^	0.67	0.28–1.63	1.15	0.55–2.42	1.16	0.54–2.52
Currently working outside the home ^c^						
3 weeks	1.03	0.25–5.20				
8 weeks			1.04	0.50–2.15		
16 weeks					1.13	0.53–2.35
Number in household	1.30	0.96–1.75	1.07	0.82–1.39	1.40	0.99–1.95
SNAP participation	2.86 *	1.13–5.21	2.33 *	1.11–4.91	2.06	0.93–4.57
WIC participation	1.64	0.60–4.49	2.38 *	1.07–5.27	2.16	0.98–4.76
Food insecurity	1.20	0.50–2.84	0.66	0.32–1.33	1.27	0.61–2.67
Infant WFLz at enrollment	1.31	0.80–2.13	1.31	0.88–1.95	0.98	0.65–1.47
**Psychosocial**						
Depressive symptoms	1.74	0.74–4.10	0.59	0.25–1.22	0.58	0.27–1.27
Relationship satisfaction	1.05	0.85–1.31	1.04	0.89–1.22	0.95	0.80–1.14
Social provision						
Reliable alliance	1.18	0.37–1.90	1.25	0.63–2.45	1.15	0.57–2.33
Guidance	1.05	0.42–2.63	0.79	0.37–1.66	1.23	0.64–2.36
Racial discrimination	2.14 *	1.10–4.17	1.31	0.71–2.42	0.89	0.40–1.94

^a^ All sociodemographic and psychosocial characteristics (except for “Currently working outside the home”) measured at infant age 1 week. ^b^ Includes “married, living together” and “involved, but not living together”. ^c^ Includes returning to “full-time” and “part-time” employment outside of the home at each time point. Reference group for the outcome variable was “cessation of any breast milk” (*n* = 123) versus “continued any breast milk” (*n* = 62) feeding at ages 3 (*n* = 26 vs. *n* = 97), 8 (*n* = 60 vs. *n* = 63), and 16 (*n* = 80 vs. *n* = 43) weeks. OR, odds ratio; CI, confidence interval; SNAP, Supplemental Nutrition Assistance Program; WIC, Special Supplemental Nutrition Program for Women, Infants, and Children; WFLz, weight-for-length z-score; * *p* < 0.05 (two-tailed), significant results are bolded for emphasis.

## Data Availability

Data used in this analysis can be obtained by contacting the corresponding author.
